# Butyrate Decreases ICAM-1 Expression in Human Oral Squamous Cell Carcinoma Cells

**DOI:** 10.3390/ijms21051679

**Published:** 2020-02-29

**Authors:** Gabriel Leonardo Magrin, Francesca Di Summa, Franz-Josef Strauss, Layla Panahipour, Michael Mildner, Cesar Augusto Magalhães Benfatti, Reinhard Gruber

**Affiliations:** 1 Department of Oral Biology, School of Dentistry, Medical University of Vienna, Sensengasse 2a, Vienna 1090, Austria; gabriel.magrin@posgrad.ufsc.br (G.L.M.); francesca.disumma@studenti.unipr.it (F.D.S.); drstrauss@odontologia.uchile.cl (F.-J.S.); layla.panahipour@meduniwien.ac.at (L.P.); 2 Center for Education and Research on Dental Implants (CEPID), Department of Dentistry, School of Dentistry, Federal University of Santa Catarina, Campus Reitor João David Ferreira Lima s/n, Florianopolis – SC 88040-900, Brazil; cesarbenfatti@yahoo.com; 3 Department of Conservative Dentistry, School of Dentistry, University of Chile, Av. Sergio Livingstone 943, Santiago 7500566, Chile; 4 Clinic of Reconstructive Dentistry, University of Zurich, 8032 Zurich, Switzerland; 5 Department of Dermatology, Medical University of Vienna, Spitalgasse 23, Vienna 1090, Austria; michael.mildner@meduniwien.ac.at; 6 Department of Periodontology, University Bern, Hochschulstrasse 4, 3012 Bern, Switzerland

**Keywords:** butyric acid, periodontium, intercellular adhesion molecule-1, oral biology, epithelial cells, in vitro

## Abstract

Short-chain fatty acids (SCFA) are bacterial metabolites that can be found in periodontal pockets. The expression of adhesion molecules such as intercellular adhesion molecule-1 (ICAM-1) within the epithelium pocket is considered to be a key event for the selective transmigration of leucocytes towards the gingival sulcus. However, the impact of SCFA on ICAM-1 expression by oral epithelial cells remains unclear. We therefore exposed the oral squamous carcinoma cell line HSC-2, primary oral epithelial cells and human gingival fibroblasts to SCFA, namely acetate, propionate and butyrate, and stimulated with known inducers of ICAM-1 such as interleukin-1-beta (IL1β) and tumor necrosis factor-alfa (TNFα). We report here that butyrate but not acetate or propionate significantly suppressed the cytokine-induced ICAM-1 expression in HSC-2 epithelial cells and primary epithelial cells. The G-protein coupled receptor-43 (GPR43/ FFAR2) agonist but not the histone deacetylase inhibitor, trichostatin A, mimicked the butyrate effects. Butyrate also attenuated the nuclear translocation of p65 into the nucleus on HSC-2 cells. The decrease of ICAM-1 was independent of Nrf2/HO-1 signaling and phosphorylation of JNK and p38. Nevertheless, butyrate could not reverse an ongoing cytokine-induced ICAM-1 expression in HSC-2 cells. Overall, these observations suggest that butyrate can attenuate cytokine-induced ICAM-1 expression in cells with epithelial origin.

## 1. Introduction

Oral health requires the cellular immunity of the oral mucosal barrier that extends towards the periodontium to defend the tooth-bearing tissue against commensal microbes and other antigens of the oral cavity [1]. The junctional epithelium controls the transmigration of neutrophils towards the crevicular fluid by means of a tightly controlled expression of adhesion molecules, thereby defending microbiological antagonism in the periodontal tissue [2,3]. Thus, the increase of adhesion molecules by inflammatory mediators has to be counterbalanced by local cues to control an excessive influx of cells of the innate immune system.

The influx of cells is controlled by intercellular adhesion molecule-1 (ICAM-1), allowing the transmigration of leucocytes which express the corresponding lymphocyte function-associated antigen-1 and macrophage adhesion ligand-1 [4]. ICAM-1, being induced by inflammatory cues such as interleukin-1-beta (IL1β) and tumor necrosis factor-α (TNFα) [5], is expressed by the vascular endothelium and by the junctional epithelium [6], thus, facilitating transmigration of leukocytes across vascular endothelia and the invasion of the extracellular matrix [7]. Although ICAM-1 is consistently expressed by junctional epithelial cells in healthy gingiva and in pocket epithelium, it is not detectable on the majority of keratinocytes in the external gingival epithelium [6,8]. The question then arises, how is the expression of ICAM-1 in epithelial cells controlled?

The increase of ICAM-1 expression by inflammatory cues is evidently well-documented. Inflammatory mediators including IL-6 and prostaglandin E_2_ increase ICAM-1 expression in human oral squamous cell carcinoma SCC4 cells in vitro [9,10]. Primary gingival epithelial cells increasingly express ICAM-1 upon inflammatory cytokines stimuli, namely, TNFα and interferon-γ [11]. Gingival fibroblasts also express ICAM-1 in response to inflammatory cytokines [12]. Nevertheless, the opposite effect, the down-regulation of ICAM-1, has not been conclusively defined. Down-regulation of ICAM-1 on bronchial epithelial cells has been observed with fenoterol, a β2-adrenoceptor agonist [13], and in retinal pigment epithelial cells with bezafibrate, a drug to treat hyperlipidemia [14]. However, little is understood about what decreases ICAM-1 expression in oral epithelial cells. 

Short-chain fatty acids (SCFA) are mainly produced by Gram-negative bacteria, being acetate, propionate, and butyrate the three most common molecules [15]. SCFA are found in the oral cavity, particularly in dental plaque and sites with periodontal disease [16,17]. Millimolar concentration of butyrate in the gingival crevicular fluid were correlated with gingival inflammation and periodontal pocket depth [18]. Anaerobic bacteria in subgingival plaques such as *Porphyromonas gingivalis, Treponema denticola, Aggregatibacter actinomycetemcomitans, Prevotella intermedia* and *Fusobacterium nucleatum* release SCFA, including butyrate [17]. Furthermore, butyrate from oral environment can cross the gingival barrier and potentially cause systemic inflammation and localized detrimental effects in the brain [19]. Taken together, it seems that butyrate and other SCFA are virulence factors in periodontal disease. 

Butyrate can activate the free fatty acid receptor-2 (FFAR2), also known as G-protein coupled receptor-43 (GPR43) [20], but also inhibit the histone deacetylase (HDAC) [21]. Using either of these mechanisms, butyrate reduces proliferation and induces apoptosis in gingival fibroblast [22,23,24,25], stimulates T-cell apoptosis [26] and osteoblast maturation [27], as well as pro-inflammatory cytokine release by neutrophils [28]. Butyrate also reduced integrin expression in Ca9-22 epithelial cells [23,29] and promoted autophagy [30]. The presence of SCFA in the infectious site attenuates the neutrophils response to *A. actinomycetemcomitans* as a result of the inhibition of specific isoforms of HDACs, namely, HDAC 1 and 3, but not activation of FFAR2 [31]. Recent findings suggest that butyrate disturbs gingival epithelial homeostasis and initiates expression of pro-inflammatory cytokine in vitro [32]. Thus, there is accumulating evidence suggesting that SCFA has detrimental effects on cells of the periodontium. However, with respect to the beneficial effects of butyrate on colitis [33,34], pathological bone loss [35], anti-microbial activity [36], and on a M1-to-M2 shift in macrophages [37,38,39] it should not be ruled out that SCFA may also contribute to tissue homeostasis by modulation of ICAM-1.

Butyrate markedly reduces ICAM-1 expression in the intestine of severely burned rats [40] and in IL1β-stimulated chondrocytes [41]. Butyrate also reduces the expression of ICAM-1 in LPS-stimulated mouse glomerular mesangial and Caco-2 cells [42,43], and cytokine-induced ICAM-1 expression in cultured endothelial cells [44]. Conversely, other studies showed that butyrate increases ICAM-1 in human gingival carcinoma cell line Ca9-22 [23,45], in acute myeloid leukemia cells [46] and endothelial cells [47,48]. Owing to these inconsistent results, it cannot be predicted whether butyrate or other SCFA change the expression of ICAM-1 in oral epithelia cells. The aim of this study was thus to investigate the influence of SCFA on the expression of ICAM-1 in oral cells with epithelial origin and to unravel possible underlying signaling pathways.

## 2. Results

### 2.1. Cell Viability Upon SCFA Stimulation at Varying Concentrations

In order to evaluate the impact of SCFA on cell viability, an MTT assay, reflecting the NAD(P)H-dependent formazan production, was carried out. To this end, HSC-2 and gingival fibroblasts were exposed to different concentration of SCFA ranging from 1 mM to 100 mM (Table 1). In case of acetate and propionate a concentration from 1 to 10 mM did not affect the viability of HSC-2 and gingival fibroblasts (Table 1). With respect to butyrate, a concentration up to 30 mM was tolerated by both cell types without altering their viability. Together, these observations indicate that 10 mM of SCFA is non-cytotoxic and therefore a suitable concentration for the following experiments. 

### 2.2. Butyrate but Not Acetate and Propionate Decrease the Expression of ICAM-1 in HSC-2 Cells

**Figure 1:**
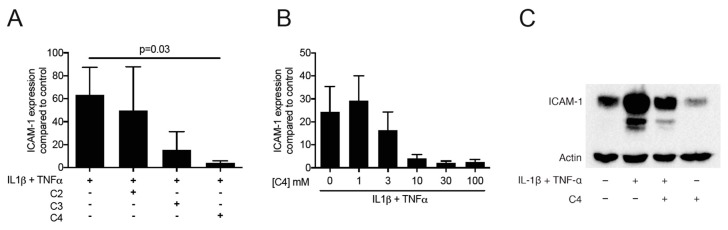
(**A**) Butyrate suppresses the cytokine-induced expression of ICAM-1 in HSC-2 cells. HSC-2 were exposed for 24 h to 10 mM of acetate (C2), propionate (C3) and butyrate (C4), and then stimulated for three hours with 10 ng/mL of IL1β and TNFα. (+), indicates presence; (−), indicates absence. Data represent the mean change of ICAM-1 expression ± standard deviation. *n* = 3. Statistical analysis was based on ANOVA test with Tukey’s multiple comparisons correction and significant *p*-values are indicated. (**B**) Butyrate suppresses the cytokine-induced increase of ICAM-1 in a dose-dependent manner. HSC-2 cells were exposed to different concentrations of butyrate in the presence of IL1β and TNFα. Data represent the mean change of ICAM-1 expression ± standard deviation. *n* = 3 (**C**) Butyrate attenuates the IL1β- and TNFα-induced expression of ICAM-1. HSC-2 cells were exposed to 10 mM of butyrate (C4) in the presence or absence of 10 ng/mL IL1β and TNFα.

**Figure 2:**
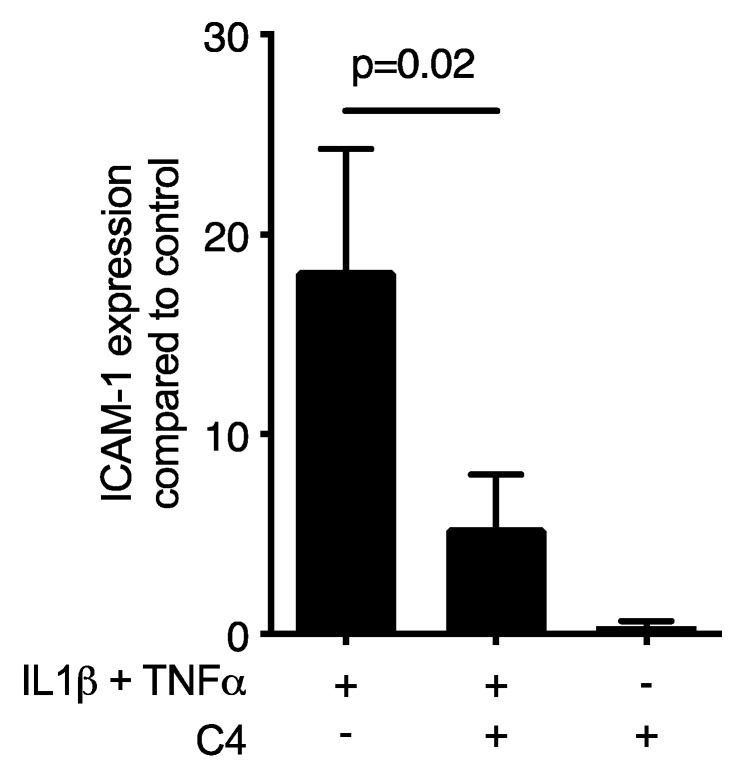
Butyrate suppresses the cytokine-induced increase of ICAM-1 in primary epithelial cells. Cells were exposed to 10 mM butyrate (C4) in the presence of 10 ng/mL IL1β and TNFα. (+), indicates presence; (−), indicates absence. Data represent the mean change of ICAM-1 expression ± standard deviation. *n* = 3. Statistical analysis was based on *t*-test and *p*-values are indicated.

**Figure 3:**
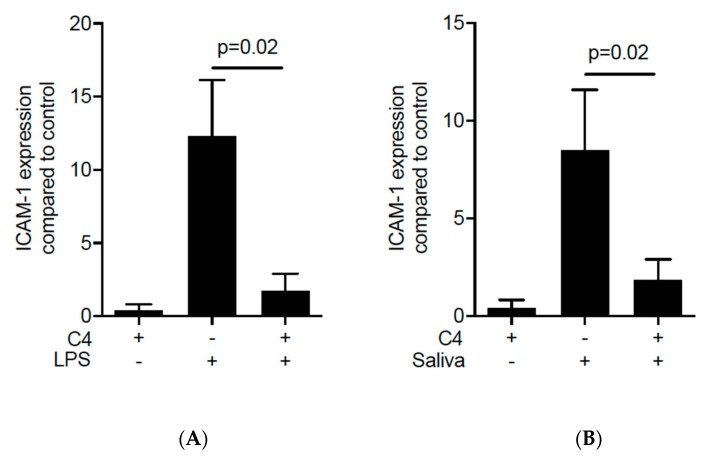
Butyrate suppresses the LPS- and saliva-induced increase of ICAM-1 in primary mouse macrophages. Cells were exposed to 10 mM of butyrate (C4) in the presence of (**A**) 100 ng/mL of LPS and (**B**) 2% saliva. (+), indicates presence; (−), indicates absence. Data represent the mean change of ICAM-1 expression ± standard deviation. *n* = 4. Statistical analysis was based on Mann–Whitney U test and *p*-values are indicated.

Then, to examine the possible role of SCFA on ICAM-1 expression, the oral squamous cell carcinoma cell line HSC-2 and gingival fibroblasts were cultured for 12 h with and without acetate, propionate and butyrate. Subsequently, the cells were exposed for three hours to known inducers of ICAM-1, namely IL1β and TNFα. Butyrate exposure at 10 mM dampened down the robust cytokine-induced ICAM-1 mRNA expression in HSC-2 cells (*p* = 0.03; Figure 1A) but not in gingival fibroblasts (Figure S1) or TR146 cells (Figure S2). In HSC-2 cells this suppression was dose-dependent (Figure 1B) and independent of the type of cytokine (Figure S3). Acetate and propionate at 10 mM, however, failed to cause a significant suppression of IL1β- and TNFα-induced ICAM-1 expression (*p* > 0.05, Figure 1A). Western blot analysis confirmed the marked suppression of ICAM-1 by butyrate (Figure 1C). Similarly, butyrate suppressed the cytokine-induced expression of ICAM-1 in primary oral epithelial cells (Figure 2). Then, and in order to validate these observations, we used another experimental setting using primary mouse macrophages [37,38,39]. Notably, butyrate was capable of inhibiting the LPS- and saliva-induced ICAM-1 expression in primary mouse macrophages (Figure 3). Collectively, these results suggest that butyrate suppresses the robust cytokine-induced ICAM-1 expression in HSC-2, primary oral epithelial cells and macrophages.

### 2.3. Activation of FFAR2 Can Mimic the Activity of Butyrate on ICAM-1 in HSC-2 Cells

**Figure 4:**
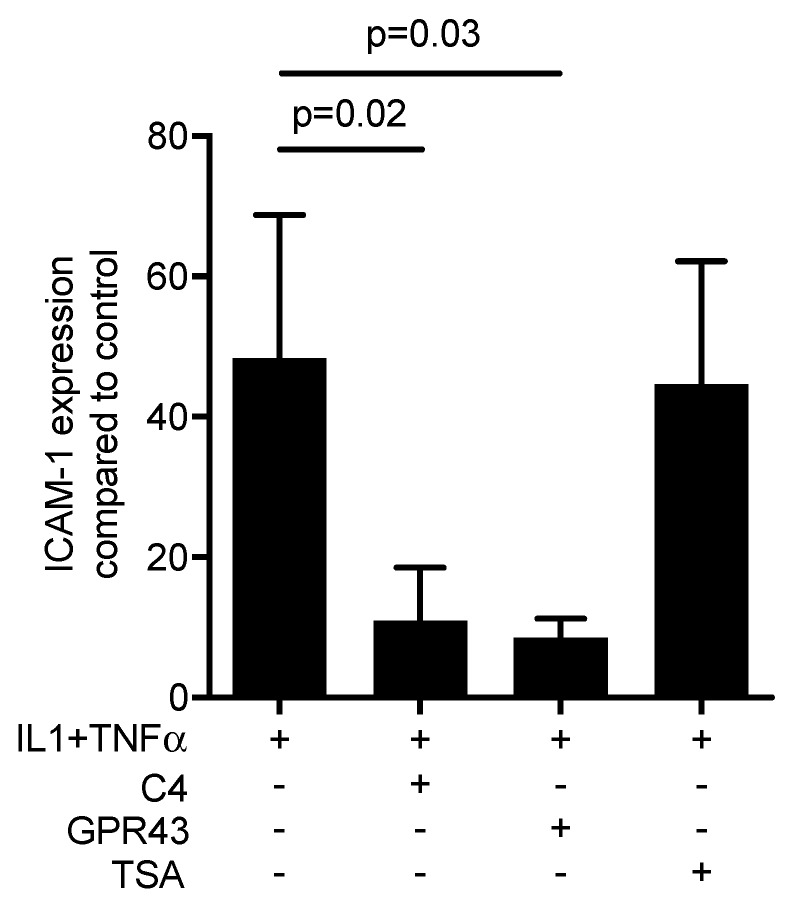
Activation of free fatty acid receptor-2 (FFAR2/GPR43) can mimic the activity of 10 mM of butyrate (C4) on ICAM-1 in HSC-2 cells. HSC-2 cells were exposed to a FFAR2/GPR43 agonist at 30 µM and trichostatin A (TSA) at 10 nM, respectively, for 24 h before ICAM-1 was induced. (+), indicates presence; (−), indicates absence. Data represent the mean change of ICAM-1 expression ± standard deviation. *n* = 3. Statistical analysis was based on Mann–Whitney U test and t-test, *p*-values are indicated.

To distinguish between the dual activity of butyrate to activate the SCFA receptor FFAR2/GPR43 [20] from the histone deacetylase inhibition [21], HSC-2 cells were pre-exposed to a FFAR2/GPR43 agonist and to trichostatin A, an inhibitor of histone deacetylases. After 24 h of stimulation, IL1β and TNFα were added to induce ICAM-1. Similar to butyrate, the FFAR2/GPR43 agonist significantly reduced the cytokine-induced expression of ICAM-1 in HSC-2 cells (Figure 4). In contrast, trichostatin A failed to inhibit the ICAM-1 expression. Surprisingly, the FFAR2 agonist GLPG 0974 failed to reverse the effects of butyrate on ICAM-1 expression (data not shown). Overall, these observations partially suggest that the suppression activity of butyrate is due to an activation of FFARs rather than an inhibition of the histone deacetylase activity.

### 2.4. Butyrate Inhibits the Nuclear Translocation of p65 on HSC-2 cells

**Figure 5:**
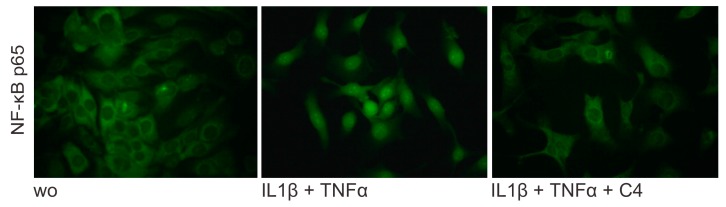
Butyrate suppresses NF-κB p65 translocation in inflamed HSC-2 cells. Butyrate at 10 mM attenuates the intracellular translocation of NF-κB p65 into the nucleus, induced by 10 ng/mL IL1β and TNFα in HSC-2. (wo), without. Representative immunofluorescence at 40x.

To explore a possible role of butyrate in inflammation, NF-kB p65 immunofluorescence was performed [49]. The presence of 10 mM of butyrate inhibited the IL1β- and TNFα-induced translocation of p65 protein into the nucleus in HSC-2 cells (Figure 5). Moreover, 10 mM of butyrate caused a 4-fold increase of β-arrestins-2 [50], known to be activated by FFAR2 receptor signaling, producing anti-inflammatory effects by inhibition of NF-κB [51,52]. This observation implies a suppression of the inflammatory activation by transcription factors triggered via NF-kB proteins that might involve Nrf2-HO1 signaling. However, blocking HO1 activity with SnPP failed to reverse the effects of butyrate on ICAM-1 expression in HSC2 cells (data not shown). Consistently, butyrate had no impact on HO-1 expression in HSC2 cells (data not shown) and the lack of Nrf2 had no impact on the suppression activity of butyrate on the LPS-induced ICAM-1 expression in primary macrophages (Figure S4). Since MAPK signaling cascade plays a key role in inflammation, we also examined the effect of butyrate on p38 and JNK expression to determine whether the suppressive activity of butyrate involves the MAPK pathway. Western blot analysis revealed that phosphorylation of p38 and JNK being involved in ICAM-1 expression, was not affected by the addition of butyrate (Figure S5). Taken together, these observations indicate that the ICAM-1-suppressive activity of butyrate is independent of Nrf2-HO1 and MAPK pathway.

### 2.5. Butyrate Cannot Reverse the Acute Expression Levels of ICAM-1 in HSC-2 Cells

**Figure 6:**
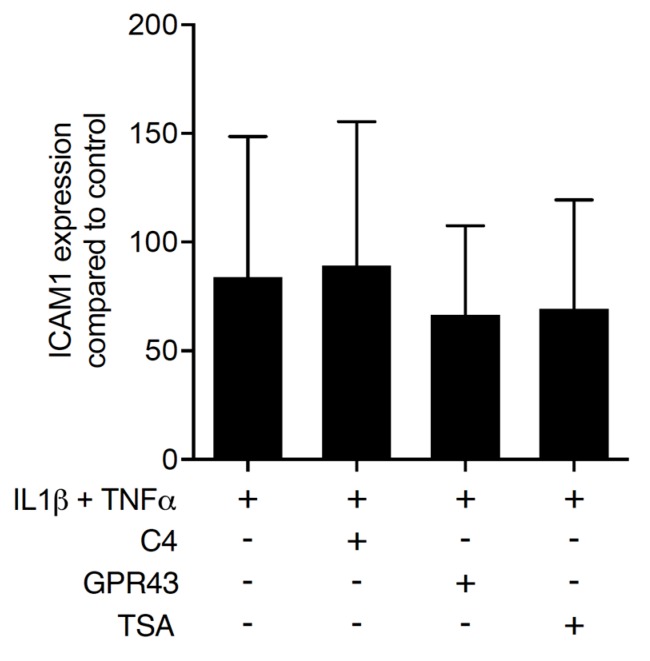
Butyrate cannot reverse expression levels of ICAM-1 in HSC-2 cells. HSC-2 cells were exposed to 10 ng/mL IL1β and TNFα for 1 h before 10 mM of butyrate (C4) was added for another 3 h. In this setting, butyrate FFAR2/GPR43 agonist and trichostatin A (TSA) failed to modulate the ICAM-1 expression in HSC-2 cells. (+), indicates presence; (−), indicates absence. Data represent the mean change of ICAM-1 expression ± standard deviation. *n* = 3. Statistical analysis was based on *t*-test.

In the previous experimental setting, HSC-2 cells were exposed to butyrate for 24 h, before the incubation with IL1β and TNFα, simulating a prophylactic effect of butyrate. To investigate the possible role of butyrate in dampening the acute ICAM-1 expression, HSC-2 cells were exposed to IL1β and TNFα for 1 h and then butyrate was added for another 3 h. In this setting, butyrate failed to modulate the ICAM-1 expression in HSC-2 cells suggesting that butyrate can prevent but not reverse an ongoing ICAM-1 expression in inflammatory conditions (Figure 6). Moreover, three hours of pre-exposure of HSC-2 cells with butyrate also failed to reduce the IL1β and TNFα-induced increase of ICAM-1, suggesting that a longer period of pre-incubation with butyrate is critical to obtain aICAM-1 modulation [53].

## 3. Discussion

Our findings showed that butyrate but not acetate or propionate attenuates the cytokine-induced ICAM-1 expression in oral squamous cells. This effect was consistent in all experiments performed, being confirmed at the transcriptional and the protein levels. The changes of ICAM-1 expression were further confirmed with primary oral epithelial cells and macrophages, while gingival fibroblasts failed to respond to butyrate. These observations raise the hypothesis that butyrate can modulate epithelial cell responses in the inflamed periodontium and thereby possibly influencing the ICAM-1-dependent transmigration of leucocytes and immune cells. It seems reasonable to relate the production of butyrate by periodontal pathogens with the severity of periodontitis. In this sense, a possible mechanism might be that the reduction of ICAM-1 expression lowers the influx of leukocytes to the inflamed tissue thereby hampering the immune system to tackle bacterial invasion. Nevertheless, further research is required to elucidate the precise role of butyrate in the periodontal tissues under different in vitro conditions.

Our research supports the role of butyrate to reduce ICAM-1 expression as observed in the intestine of burned rats [40], stimulated chondrocytes [41], glomerular mesangial cells [42], colon cancer cells [43], and endothelial cells [44]. These observations together with our findings are, however, in contrast to those showing that butyrate increases ICAM-1 in gingival carcinoma cells [23,45], leukemia cells [46] and endothelial cells [47,48]. Furthermore, high concentrations of butyrate provoke apoptosis in inflamed human gingival fibroblasts and periodontal destruction [25,29]. The testing of acetate and propionate was also hampered by the higher toxicity compared to butyrate. Surprising was that although acetate and propionate are agonist for the FFAR2/GPR43, only butyrate caused the robust and significant suppression of ICAM-1 expression [52]. Moreover, blocking of FFAR2/GPR43 by the antagonist GLPG 0974 failed to reverse the effects of butyrate on ICAM-1 expression. This raises the question whether the FFAR3/GPR41 is mediating the activity of butyrate [54]. Thus, further research is necessary to unravel the underlying mechanism at the receptor level.

The FFAR2 receptor activates β-arrestins-2, producing anti-inflammatory effects by inhibition of NF-κB [51,52] and ICAM-1 has a NFκB binding prompter region [55]. In support of this potential mechanism, we show that β-arrestins-2 is increased by butyrate in HSC-2 cells. Hence, the blocking of NFκB nuclear translocation likely reduces ICAM-1 expression, being in line with our main observations on the regulation of ICAM-1 by butyrate. The reason why TR146 cells and gingival fibroblasts failed to respond to butyrate may be explained by their low increase of ICAM-1 expression in response to inflammatory cytokines. However, also short-time exposure of HSC-2 cells to butyrate had no considerable effect suggesting that not enough β-arrestins-2 is produced to reduce NF-κB signaling. Certainly, the role of butyrate to change β-arrestins-2 expression and the involvement in ICAM-1 expression in HSC-2 cells should be investigated. Although many questions remain open, the present data clearly show that butyrate can prevent the cytokine-induced ICAM-1 expression in oral squamous cell carcinoma cells, primary oral epithelial cells and macrophages.

To better understand the underlying molecular mechanisms, the Nrf2-HO1 pathway was investigated. Butyrate uses the Nrf2/HO-1 pathway to ameliorate diabetic nephropathy [56], to regulate Th17/Treg cell balance [57] and to protects against high-fat diet-induced oxidative stress in rat liver [58]. There is also evidence that butyrate inhibits the acute lung injury in mice by regulating the NFκB signaling pathway [59]. Moreover, Nrf2-HO1 signaling is linked to ICAM-1 expression in a mouse atherosclerosis model [60], in THP-1 macrophages [61], and HaCaT cells [62]. However, in the present study, SnPP blocking HO1 activity could not reverse the inhibition of butyrate in ICAM-1 expression. Indeed, macrophages from Nrf2 knockout and wildtype mice showed similar inhibition of ICAM-1 expression. Even though Nrf2-HO1 signaling was a strong candidate to mediate the effects of butyrate, this mechanism is presumably not relevant for the observations we have reported here. Furthermore, butyrate failed to reduce the phosphorylation of p38 and JNK, both major signaling molecules driving NFκB signaling and ICAM-1 expression in HSC-2 cells suggesting that other pathways than Nrf2-HO1 and MAPK signaling are relevant to explain the strong inhibition of ICAM-1 by butyrate [62,63,64].

Are the present findings clinically relevant? To answer that question, it is worth mention that the commensal bacteria that induce a low-grade inflammatory state in the junctional epithelium are likely the triggers of ICAM-1 expression and neutrophils migration [7]. Thus, there is strong ICAM-1 staining of the junctional epithelium in both clinically healthy and inflamed tissue [2], even though soluble ICAM-1 shed into the gingival crevicular fluid was higher in patients with inflammation [63]. Considering that mice deficient in ICAM-1 have impaired immune function and decreased inflammatory response [64], a decrease of ICAM-1 in epithelial cells caused by butyrate might weaken the innate immunity and thus the local defense of the periodontium. Regarding the role of ICAM-1 in macrophages, this also remains controversial. ICAM-1 deficiency increases M2 macrophage polarization and suppress tumor metastasis [65], but others reported that downregulation of ICAM-1 in RAW264.7 macrophages resulted in inflammatory M1 polarization [66]. Therefore, the possible implication of ICAM-1 expression in macrophages and its regulation by butyrate in periodontal tissue homeostasis remains to be determined.

This study has limitations that need to be acknowledged. Our finding that butyrate protects cells from cytokine-induced ICAM-1 expression in oral squamous cell carcinoma cells in vitro does not necessarily explain the in vivo situation. Notably, butyrate failed to diminish an ongoing ICAM-1 expression in HSC-2 cells. It would be interesting to determine the impact of SCFA on periodontitis by means of in vivo models. For example, it can be suggested to study the role of epithelial ICAM-1 on healthy periodontium, the impact of the SCFA receptors in this context, and whether butyrate produced by periodontal pathogens plays a role in pathogenesis of periodontitis. Furthermore, exciting questions about how SCFA from non-digestible nutritional fibers metabolized by gut bacteria [67] can enter the bloodstream and elicit systemic effects should be addressed. An optimized diet rich in fibers can reduce gingival and periodontal inflammation in humans [68,69]. This observation inspires further research towards a possible beneficial role of butyrate on ICAM-1 expression in the periodontal tissue. Moreover, future studies should be performed in FFAR2 and FFAR3-deficient mice asking if butyrate can prevent inflammatory osteolysis [70,71], and if yes, if this effect also involves the blocking of the histone deacetylase [72,73].

In conclusion, our findings that butyrate modulates the inflammatory response in oral epithelial cells by decreasing ICAM-1 expression provide a new step towards understanding the effect of ICAM-1 under inflammatory conditions. These in vitro data may inspire future research on the mechanisms by which diet, microbiota and other factors influence the immune system and, consequently, the development of inflammatory and infectious diseases.

## 4. Material and Methods

### 4.1. Cell Culture

Human oral epithelial carcinoma cells HSC-2 and TR146 were kindly provided by Prof. Rausch-Fan from Medical University of Vienna, Vienna, Austria. Gingival fibroblasts and primary epithelial cells were obtained from human gingiva harvested from extracted third molars of patients who had given informed and written consent. Gingival fibroblasts were prepared by explant cultures. The epithelium was separated from the underlying connective tissue after overnight dispase II (2.4 U/mL; Roche, Mannheim, Germany) treatment, and preparation of single-cell suspension by means of trypsin (Lonza, Walkersville, MD) digestion at 37 °C for 10 min. Epithelial cells were expanded in growth medium-2 (KGM-2; Lonza, Basel, Switzerland). The Ethics Committee of the Medical University of Vienna (EK NR 631/2007, 19 March 2019) Vienna, Austria, approved this protocol. Cell lines and gingival fibroblasts were cultured in Dulbecco’s modified Eagle medium (DMEM, Invitrogen Corporation, Carlsbad, CA, USA) supplemented with 10% fetal calf serum (Invitrogen Corporation, Carlsbad, CA, USA) and antibiotics (Invitrogen Corporation, Carlsbad, CA, USA) at 37 °C, 5% CO_2_, and 95% humidity. Gingival epithelial cells were cultured in keratinocyte growth medium-2 (KGM-2; Lonza, Basel, Switzerland) at 37 °C, 5% CO_2_, and at 95% relative humidity. Cells were seeded in growth medium at a concentration of at least 30,000 cells/cm^2^ onto culture dishes one day prior to stimulation. Serum-free conditions were used during cell stimulation. For the isolation and culture of murine bone marrow-derived macrophages, BALB/c mice (Animal Research Laboratories, Himberg, Austria) of 6–8 weeks old were purchased. Bone marrow cells were collected from the femora and tibiae and grown for 5 days in Minimum Essential Medium Eagle-Alpha Modification (αMEM, Invitrogen Corporation, Carlsbad, CA, USA), supplemented with 10% fetal calf serum and antibiotics, supplemented with 20 ng/mL macrophage colony-stimulating factor (M-CSF; ProSpec-Tany TechnoGene Ltd., Rehovot, Israel). For selected experiments, cells from Nrf2 knockout mice and the respective wildtype controls were used (Prof. Florian Gruber, Department of Dermatology, Medical University of Vienna, Vienna, Austria).

### 4.2. Viability Assay

Epithelial HSC-2 cells and gingival fibroblasts were incubated with different concentrations of acetate, propionate and butyrate (Sigma-Aldrich, St. Louis, MO, USA) or serum-free medium in 96-well plates (CytoOne, Starlab International, Hamburg, Germany). After 24 h, a final concentration of 0.5 mg/mL of a MTT - 3-(4,5-dimethythiazol-2-yl)-2,5-diphenyltetrazolium bromide – (Sigma-Aldrich, St. Louis, MO, USA) solution was added to each well of the microtiter plate for 3 h at 37 °C. After medium removal, formazan crystals were solubilized with dimethyl sulfoxide. Assessment of optical density was carried out for 570 nm. Absolute numbers of optical density in the treatment groups were expressed and presented as percentage of unstimulated controls ± standard deviation.

### 4.3. Cell Stimulation

Based on the findings from the viability assay, stimulation of HSC-2 cells and gingival fibroblasts was performed with 10 mM of acetate, propionate and butyrate. As a basic setting for the experiments, cells were exposed to SCFA for 24 h in serum-free medium before the addition of 10 ng/mL IL1β and TNFα (ProSpec-Tany TechnoGene Ltd., Rehovot, Israel) for another three hours, after that gene expression analysis of ICAM-1 was performed. Likewise, the oral squamous cell carcinoma cell line TR146 was exposed to butyrate in the same conditions of HSC-2. Macrophages were exposed to SCFA for 24 h in growth medium before the addition of LPS (100 ng/mL, LPS from Escherichia coli 0111: B4; Sigma-Aldrich, St. Louis, MO, USA) or 2% of sterile pooled human saliva [74] for another three hours. For dose-response experiments in HSC-2 cells, concentrations of 1, 10, 30 and 100 mM of butyrate were used. HSC-2 cells were further exposed to GPR43 (FFAR2) agonist (Merck KGaA, Darmstadt, Germany) at 30µM and the histone deacetylase inhibitor trichostatin A at 10 nM (Sigma-Aldrich, St. Louis, MO, USA) for 24 h before inflammation was induced accordingly. In another series of experiments, HSC-2 cells were exposed to IL1β and TNFα for 1 h followed by the exposure to 10 mM butyrate for three hours, or exposed to butyrate either alone or in the presence of tin protoporphyrin IX dichloride (SnPP; Sigma-Aldrich, St. Louis, MO, USA) at a concentration of 10 µM. We have included a rescue experiment by using the inhibitor of FFAR2 named GLPG 0974 at 10µM (Tocris Bioscience™, Abingdon, UK).

### 4.4. qRT-PCR Analysis

ExtractMe total RNA kit (Blirt S.A., Gdańsk, Poland) was used for RNA isolation. Reverse transcription was then performed by means of SensiFAST^TM^ cDNA (Bioline, London, UK). For polymerase chain reaction, SensiFAST^TM^ SYBR ROX Kit (Bioline, London, UK) on a Real-Time PCR Detection System (Bio-Rad Laboratories, Hercules, CA, USA) was carried out. Primer sequences used are described in Table 2. The mRNA levels were calculated by normalizing to the housekeeping gene GAPDH using the ΔΔCt method.

### 4.5. Western Blot

After stimulation with butyrate for 24 h and the inflammatory cytokines IL1β and TNFα for another three hours, HSC-2 cells were extracted with SDS buffer and inhibitors of protease (PhosSTOP with cOmplete; Sigma-Aldrich, St. Louis, MO, USA), divided by SDS-PAGE and transferred onto nitrocellulose membranes (Whatman, GE Healthcare, General Electric Company, Fairfield, CT, USA). Thereafter, membranes were submitted to blocking process for 2 h and exposed to the first antibodies (mouse ICAM-1 G-5 and actin C-2 both at 200 ng/mL; Santa Cruz Biotechnology, Santa Cruz, CA, USA) for 24 h. In another series, HSC-2 cells were exposed to butyrate for 24 h and to the cytokines for 30 min before exposed to antibodies against phosphorylated and complete p38 and c-Jun N terminal protein kinase MAPK (both Cell Signaling Technologies, Danvers, MA, USA). Then, proteins were detected by the appropriate HRP-conjugated secondary antibody at 40 ng/mL (Santa Cruz Biotechnology, Santa Cruz, CA, USA). Subsequently, chemiluminescence detection (Clarity ECL Western Blot Substrate kit, Bio-Rad Laboratories, Hercules, CA, USA) was performed with a ChemiDoc MP System (Bio-Rad Laboratories, Hercules, CA, USA).

### 4.6. Immunofluorescence

Immunofluorescent analysis was performed on HSC-2 cells plated onto Millicell^®^ EZ slides (Merck KGaA, Darmstadt, Germany) treated with 10 mM of butyrate overnight and then exposed to IL1β and TNFα for 30 min. Cells were fixed in 4% paraformaldehyde and blocked in 1% BSA and 0.1% Triton in buffered saline before being incubated with nuclear factor kappa B (NF-κB) p65 antibody (25 ng/mL, rabbit, Cell Signaling Technology, MA, USA) overnight at 4 °C. After washing, Alexa Fluor 488 secondary antibody (4 µg/mL; anti-rabbit, Cell Signaling Technology, MA, USA) was applied for 1 h at room temperature. Glass slides were mounted and images were captured at 40x under a Zeiss Axiovert 200 M fluorescent microscope (Carl Zeiss AG, Oberkochen, Germany).

### 4.7. Statistical Analysis

All experiments were repeated at least three times. Bars show the mean and standard deviation of the data from all independent experiments. Normality of the data was assessed using the Shapiro-Wilk test. Statistical analysis was based on t-test and ANOVA or Mann–Whitney U test depending on the distribution of the data. Analyses were performed using Prism v7 (GraphPad Software, La Jolla, CA, USA). Significance was set at *p* < 0.05.

## Figures and Tables

**Table 1:** Cell viability of HSC-2 and gingival fibroblasts at varying concentrations of SCFA.

Cell Type	HSC-2 Cell Line			Gingival Fibroblasts		
Concentration	Acetate	Propionate	Butyrate	Acetate	Propionate	Butyrate
100 mM	39.2 ± 5	46.4 ± 5.1	53.5 ± 5	12.5 ± 1.2	11.9 ± 1.5	10.1 ± 0.9
30 mM	45.8 ± 5.9	56.6 ± 5.8	95.8 ± 6	69.8 ± 4.5	74.5 ± 2.1	94 ± 0.5
10 mM	104.7 ± 6.1	113 ± 6.4	122.7 ± 6.5	96.3 ± 1.2	102.7 ± 1.2	122.3 ± 3.1
1 mM	125 ± 7.0	136 ± 5.6	139 ± 7.7	125.3 ± 6.7	130.4 ± 0.5	135.5 ± 6.5

HSC-2 cells and gingival fibroblasts exposed at different concentration of SFCA. Cell viability is represented by formazan production indicated in percentage of unstimulated controls ± SD. Cells maintained their viability with up to 10 mM of acetate and propionate, and up to 30 mM of butyrate.

**Table 2:** Primer sequences.

Primer	Sequence Forward	Sequence Reverse
hICAM-1	cct tcc tca ccg tgt act gg	agc gta ggg taa ggt tct tgc
hARRB2	caa ctc cac caa gac cgt caa ga	ttc gag ttg agc cac agg aca ctt
hGAPDH	aag cca cat cgc tca gac ac	gcc caa tac gac caa atc c
hActin	cca acc gcg aga aga tga	cca gag gcg tac agg gat ag
mICAM-1	gtg atg ctc agg tat cca tcc a	cac agt tct caa agc aca gcg
mGAPDH	aac ttt ggc att gtg gaa gg	gga tgc agg gat gat gtt ct
mActin	cta agg cca acc gtg aaa ag	acc aga ggc ata cag gga ca

## Supplementary Materials

Supplementary materials can be found at https://www.mdpi.com/1422-0067/21/5/1679/s1.
