# Supplementary material for: Butyrate Decreases ICAM-1 Expression in Human Oral Squamous Cell Carcinoma Cells
Source: Int J Mol Sci. 2020 Feb 29;21(5):1679. doi: 10.3390/ijms21051679 (PMC7084181; doi:10.3390/ijms21051679)
Supplement: Supplementary file 1 [file ijms-21-01679-s001.pdf]

International Journal of *Molecular Sciences*

SUPPLEMENT

Butyrate decreases ICAM-1 expression in human oral squamous cell carcinoma cells

Gabriel Leonardo Magrin* (1, 2), Francesca Di Summa* (1), Franz-Josef Strauss (1, 3), Layla Panahipour (1), Michael Mildner (4), Cesar Augusto Magalhães Benfatti (2), Reinhard Gruber (1,5)

1. Department of Oral Biology, School of Dentistry, Medical University of Vienna, Sensengasse 2a, 1190 Vienna, Austria
2. Center of Education and Research on Dental Implants, Department of Dentistry, Federal University of Santa Catarina, Florianopolis – SC, Brazil
3. Department of Conservative Dentistry, School of Dentistry, University of Chile, Av. Sergio Livingstone 943, Santiago, Chile
4. Clinic of Reconstructive Dentistry, University of Zurich, Zurich, Switzerland
5. Department of Dermatology, Medical University of Vienna, Vienna, Austria
6. Department of Periodontology, University Bern, Switzerland

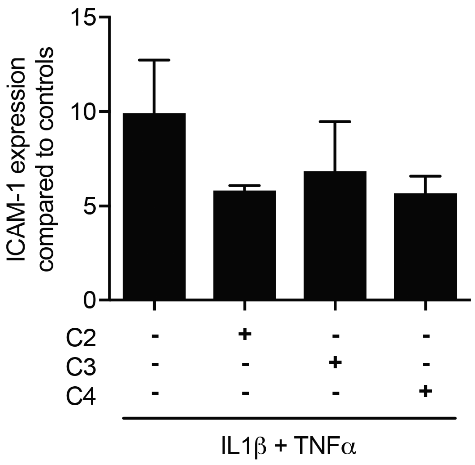

SUPPLEMENT Figure 1. Butyrate failed to suppress the increase of ICAM-1 in gingival fibroblasts. Gingival fibroblasts were exposed to 10mM acetate (C2), propionate (C3) and butyrate (C4) in the presence of 10 ng/ml IL1β and TNFα. Data represent the mean change of ICAM-1 expression ± standard deviation. N=3. Statistical analysis was based on ANOVA and Tukey`s multiple comparisons correction .

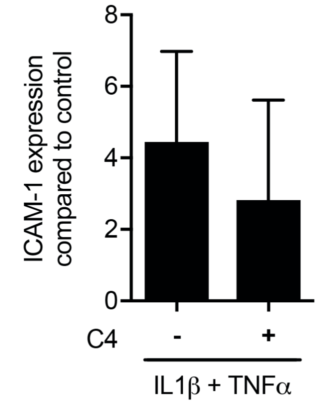

SUPPLEMENT Figure 2. Butyrate failed to suppress the cytokine-induced increase of ICAM-1 in TR146 cells.

TR146 cells were either exposed or not to 10μM butyrate (C4) in the presence of 10 ng/ml IL1β and TNFα. Data represent the mean change of ICAM-1 expression ± standard deviation. N=3. Statistical analysis based on Mann-Whitney U test.

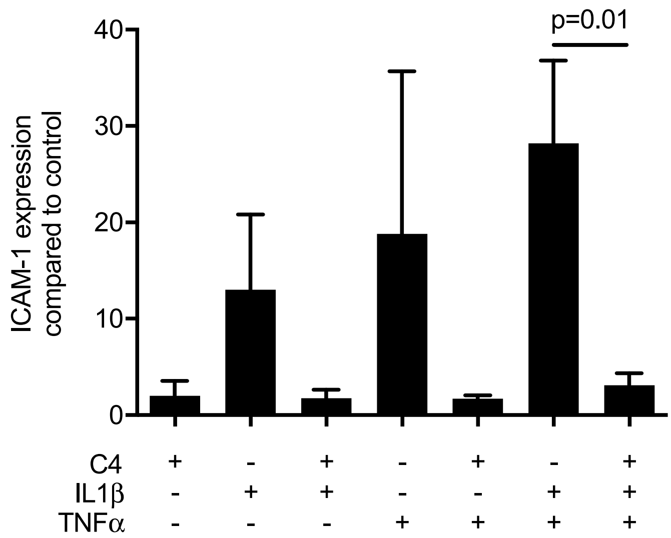

SUPPLEMENT Figure 3. Butyrate suppress the IL1β or TNFα-induced increase of ICAM-1 in HSC-2 cells.

HSC-2 cells exposed to 10mM butyrate (C4) were independently stimulated with 10 ng/ml IL1β or TNFα for three hours to push ICAM-1 expression in HSC-2 cells. Data represent the mean change of ICAM-1 expression ± standard deviation. N=3. Statistical analysis based on Mann-Whitney U test and t-test.

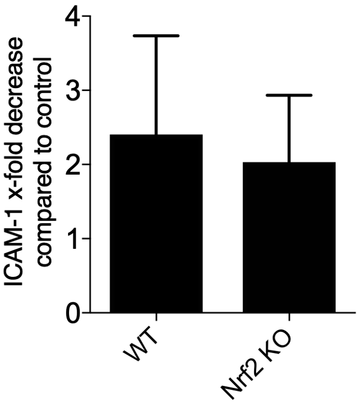

SUPPLEMENT Figure 4. Macrophages from Nrf2 knockout mice respond to butyrate decreasing ICAM-1. Macrophages from Nrf2 knockout and the respective wildtype controls mice were exposed to 10mM butyrate for 24 hours before LPS-induced ICAM-1 expression was detected. Graphs represent the relative reduction of ICAM-1 expression. N=4. Statistical analysis based on Mann-Whitney U test showed no significance.

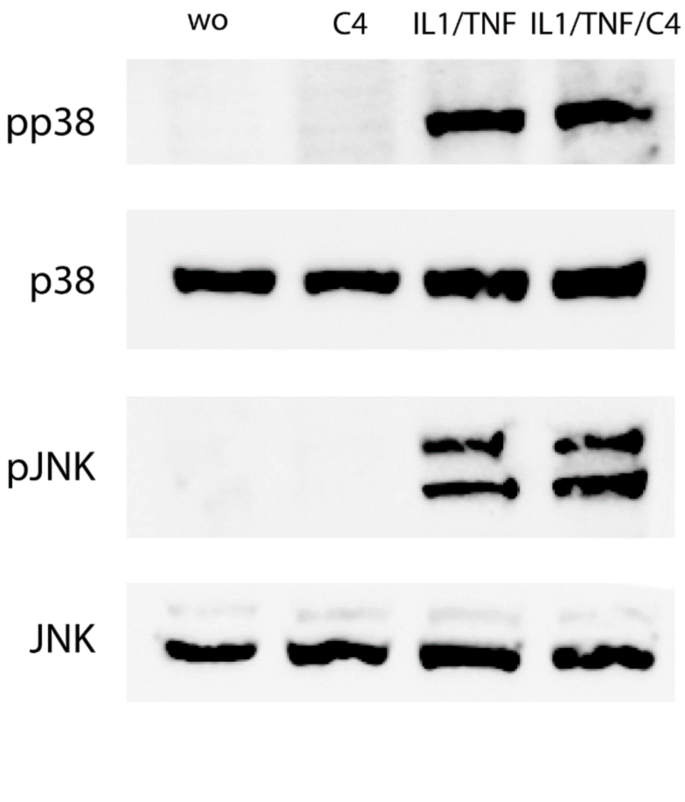

SUPPLEMENT Figure 5. Butyrate failed to change the phosphorylation of MAPK in HSC-2 cells. HSC-2 cells were exposed to butyrate (C4) for 24 hours before the phosphorylation was induced with 10 ng/ml IL1β and TNFα for 30 minutes. wo=control group. The phosphorylation levels were detected by Western blot analysis.
